# Which factors affect anxiety in rural older adults in China?

**DOI:** 10.3389/fpubh.2025.1563505

**Published:** 2025-06-11

**Authors:** Hui Wang, Chi Wang, Yi Li, Yuran Liu, Hongxu Yuan, Yujie Chen, Hong Ding

**Affiliations:** Department of Health Service Management, School of Health Management, Anhui Medical University, Hefei, Anhui, China

**Keywords:** older adults, anxiety, random forests, coarse grains consumption, visual condition

## Abstract

**Objective:**

To explore the influencing factors of individual anxiety in rural older adults in China and analyze the relative importance of each factor, so as to provide scientific basis for the development of intervention measures to improve the mental health of rural older adults. The study also aims to improve the well-being and quality of life of older adults.

**Methods:**

A multi-stage cluster random sampling was conducted in rural Anhui Province, China from July 2023 to February 2024, involving a total of 1,546 older adults. T test, ANOVA test, multiple linear regression and random forest algorithm were used for analysis.

**Results:**

The mean anxiety score among rural older adults was 4.23 ± 5.30, and the detection rate of anxiety was 36.40%. Multiple linear regression results revealed that age, marital status, visual condition, self-rated health, physical examination, consumption of coarse grains, sleep quality, subjective well-being and social support level were the influencing factors of anxiety in the older adults. The results of random forest analysis showed that depression, self-rated health, subjective well-being, sleep quality and visual acuity had great influence on anxiety in the older adults.

**Conclusion:**

This study confirmed the influence of multiple factors on the anxiety of rural older adults. In addition, the study also found that consumption of coarse grains was negatively associated with anxiety in older adults. In order to improve the mental health of the older adults in rural areas, it is recommended to pay more attention to the older adults with depression, poor sleep quality and poor eyesight, and implement multi-dimensional intervention to improve the happiness of the older adults.

## Introduction

In recent years, many countries around the world are experiencing rapid aging, which has drawn increasing attention to the physical and mental health of older adults. In terms of mental health, numerous studies have shown that more than 20% of older adults suffer from different mental disorders, with anxiety being the most prevalent (1). Anxiety is the sixth leading cause of disability globally and is a mental health condition characterized by distress and emotional discomfort (2). Some studies indicate that older adults may be more vulnerable to anxiety. Older adults often rely heavily on social factors for their economic stability, physical well-being, and mental health. Consequently, their emotional capacity to cope with social and environmental changes becomes more fragile in old age. Life circumstance alterations, shifts in social roles, and the fear of mortality are more likely to induce anxiety among older adults (3). The anxiety experienced by the older adults is predominantly manifested through physical symptoms, such as insomnia, behavioral changes, sensory disturbances, urinary issues, cardiovascular conditions, gastrointestinal disorders, and other related diseases. Anxiety has significant adverse effects on the lives of older adults, leading to a diminished quality of life, increased disability, higher demand for healthcare services, and even elevated mortality rates (4). However, a number of older adults individuals perceive emotional issues, such as anxiety, as an inevitable consequence of aging. Consequently, they do not proactively seek medical intervention or support. As a result, only a limited proportion of the older adults population has been diagnosed with anxiety and is currently receiving treatment (5). Anxiety disorders, in comparison to cognitive disorders such as chronic diseases or dementia, tend to be less effectively managed. Consequently, the early identification and preventive treatment of anxiety disorders are of critical importance. Currently, various countries and regions have carried out research on anxiety among the older adults to varying extents. Factors such as demographic characteristics (e.g., age, marital status, gender), health conditions, lifestyle behaviors (e.g., smoking, drinking), and social trust have been identified as potentially associated with anxiety (6). In summary, the risk factors associated with anxiety are multifaceted, and the relative significance of these factors remains unclear, thus necessitating further investigation. Consequently, this study aims to examine the prevalence of anxiety among the older adults in rural China, analyze potential correlates of anxiety from various dimensions, and quantify the importance of these factors using the random forest method. This research holds substantial reference value for mitigating the risk of anxiety and enhancing the mental health of the older adults population.

## Subject and methods

### Study population

From July 2023 to February 2024, a survey was conducted in Anhui Province, China, employing a multi-stage cluster random sampling method. Taking into account the variations in geographical location and regional development levels, we selected one county each from southern Anhui (Anqing), central Anhui (Hefei), and northern Anhui (Suzhou). Subsequently, 4, 10, and 4 villages were chosen, respectively, from these counties, totaling 18 villages. Face-to-face interviews were conducted with all older adults residents within these villages. The inclusion criteria were as follows: (1) age of 60 years or older; (2) clear consciousness and effective communication ability; (3) provision of informed consent and voluntary participation in the study. Exclusion criteria included: (1) cognitive impairment or inability to communicate; (2) inability to participate in the survey due to various reasons. This study adhered to the principles outlined in the Helsinki Declaration to safeguard the authenticity and confidentiality of the field investigations. A total of 1,556 older adults individuals were surveyed, yielding valid data for 1,546 participants, resulting in a final participation rate of 99.35%.

### Investigation methods

On the basis of a comprehensive review of relevant literature, we developed a structured questionnaire tailored to the life characteristics of older adults individuals in rural China. Data collection was conducted through face-to-face interviews by a team of uniformly trained investigators, all of whom were postgraduate students from Anhui Medical University. The questionnaire encompassed socio-demographic characteristics, health status, lifestyle, sleep quality, depression, social support level. Socio-demographic characteristics included age, gender, educational attainment, marital status, living arrangement, type of medical insurance, primary source of financial support, subjective well-being, physical work intensity, and results of physical examinations. Health status covered self-rated health (SRH), prevalence of chronic diseases, body mass index (BMI), and vision condition. We employed the item “If 0 represents the worst imaginable health state and 100 represents the best imaginable health state, how do you rate your health today?” from the Quality of Life Assessment Scale (EQ-5D-5L) to evaluate the self-rated health status of older adults individuals. A score of 80 or above was classified as indicating good health (7). According to the Chinese standard (8), BMI classification was conducted as follows: lean (<18.5 kg/m^2^), normal weight (18.5–23.9 kg/m^2^), overweight (24–27.9 kg/m^2^), and obesity (≥28 kg/m^2^). Lifestyle encompasses smoking habits, drinking habits, and the consumption of whole grains. The levels of anxiety, depression, social support, and sleep quality among the older adults were evaluated using the Generalized Anxiety Disorder Scale (GAD-7), Patient Health Questionnaire Depression Scale (PHQ-9), Social Support Rating Scale (SSRS), and Pittsburgh Sleep Quality Index (PSQI). These four scales demonstrate excellent applicability and have been extensively utilized in prior research on aging populations (9–11). GAD-7 is a self-administered rating scale that evaluates the frequency of symptoms experienced over the past 2 weeks. It is designed to screen for generalized anxiety disorder and assess its severity. The GAD-7 comprises seven items, each rated on a 4-point Likert scale ranging from “never” (0) to “almost every day” (3). Scores are summed to produce a total score between 0 and 21, with higher scores indicating greater levels of anxiety (12). The Patient Health Questionnaire-9 (PHQ-9) comprises nine items, each rated on a 4-point Likert scale ranging from “never = 0” to “almost every day = 3.” The total score ranges from 0 to 27, with a score of ≥5 indicating the presence of depressive symptoms (13). The Social Support Scale (SSRS) comprises three primary dimensions: subjective support, objective support, and support utilization. A Likert scale with five levels was employed, where each item was rated on a 1-to-5-point scale. Scores of ≤22 indicated low social support, scores between 23 and 44 denoted moderate social support, and scores of ≥45 reflected high social support. Higher scores corresponded to higher levels of social support (14). This questionnaire demonstrates excellent reliability and validity and has been extensively utilized in research involving the older adults population in China (15, 16). The Pittsburgh Sleep Quality Index (PSQI) is a validated tool that can be utilized to assess the sleep quality of older adults individuals over the past month. This scale comprises seven dimensions, each of which is rated on a scale from 0 to 3. A PSQI score exceeding 7 indicates poor sleep quality, whereas a score below 7 suggests good sleep quality. Notably, higher PSQI scores are associated with poorer sleep quality (17).

### Quality control

During the investigation, the researcher endeavors to provide detailed explanations of the questions to respondents using clear verbal communication. Upon completion of the investigation, the researcher retains the paper questionnaire for record-keeping purposes. Prior to respondents’ departure, the researcher conducts a thorough review to identify any missing items, incorrect entries, or logical inconsistencies, allowing for immediate corrections. The data entry process undergoes rigorous scrutiny to ensure the accuracy and reliability of the collected information.

### Random forest regression

Random forest is a machine learning algorithm that leverages an ensemble of classification trees to address both classification and regression tasks. This model employs bootstrap repeated sampling to extract multiple subsets from the original dataset, constructs a decision tree for each subset, and subsequently integrates the predictions of these trees via voting to produce the final prediction outcome (18). Random forest regression is a non-parametric regression technique that can effectively analyze collinear, non-linear, and interactive data. Unlike traditional regression methods, it does not require the pre-specification of a hypothetical model for the relationship between independent and dependent variables. Instead, it continuously trains on acquired information to construct multiple “decision trees,” forming a “random forest.” This ensemble approach is then used to regress samples and output the importance of each independent variable’s effect on the dependent variable (19). In recent years, the random forest algorithm has gained widespread application in predicting disease risk models and analyzing disease risk factors among the older adults population (20–22). Given that the anxiety score of the dependent variable in this study is a continuous variable and there is a need to measure the importance of the independent variables, a random forest regression model was constructed.

### Statistics analysis

We utilized Epidata 3.1 for data entry and SPSS 26.0 for statistical analysis. The mean and standard deviation were employed to describe the scores. A *T*-test and ANOVA test were conducted for univariate analysis, while multiple linear regression was performed to examine the influencing factors of anxiety among the rural older adults population. Statistical significance was determined at *p* < 0.05. Additionally, RStudio software was used to implement random forest algorithm analysis, and the importance of the influencing factors was ranked accordingly.

## Results

Subject general characteristic.

A total of 1,546 rural older adults individuals were included in this study. Among them, 537 were aged 60–69 years, and 743 were aged 70–79 years. The sample consisted of 838 women. Regarding marital status, data were available for 1,151 participants. Additionally, 883 participants were illiterate, and 1,243 were non-cohabiting older adults individuals. A total of 1,485 older adults individuals participated in the urban and rural residents’ medical insurance program. Furthermore, 1,145 participants reported having chronic diseases, and 805 experienced blurred vision. Smoking and alcohol consumption data indicated that 1,131 were non-smokers and 1,072 did not consume alcohol. Depression was identified in 648 older adults, while only 53 older adults reported low levels of social support.

### Anxiety scores of rural older adults with different characteristics

The average anxiety score among the rural older adults was 4.23 ± 5.30, with 564 individuals experiencing anxiety, resulting in a detection rate of 36.40%. Univariate analysis revealed significant differences in anxiety scores based on age, sex, marital status, education level, primary income source, subjective well-being, presence of chronic diseases, visual acuity, smoking habits, alcohol consumption, labor intensity, health examination participation, social support, depression levels, sleep quality, consumption of coarse grains, BMI levels, and self-rated health (*p* < 0.05). For detailed results (see Table 1).

**Table 1:** Anxiety scores of rural older adults with different characteristics.

Variable	*n*	Anxiety score	*t*/*F*	*P*
Age			4.96	*P <* 0.05
60–69	537	3.80 ± 4.84		
70–79	743	4.67 ± 5.69		
80 and above	266	3.87 ± 4.95		
Gender			−6.81	*P* < 0.05
Male	708	3.26 ± 4.63		
Female	838	5.05 ± 5.67		
Marital status			1.97	*P* < 0.05
Married	1,151	4.38 ± 5.45		
Not married	395	3.81 ± 4.79		
Educational level			6.17	*P* < 0.05
Illiterate	883	4.91 ± 5.88		
Literate	663	3.32 ± 4.22		
Residential status			1.56	*p* = 0.12
Live alone	303	3.81 ± 5.12		
Not living alone	1,243	4.33 ± 5.34		
Medical insurance type			0.80	*p* = 0.44
Urban and rural residents’ Medical insurance	1,485	4.23 ± 5.32		
Urban employee Medical Insurance	45	4.73 ± 5.03		
Other	15	2.73 ± 2.58		
Primary source of financial support			8.37	*P* < 0.05
Self-labor	585	3.83 ± 5.09		
Child support	549	3.85 ± 4.84		
Government subsidies	323	5.70 ± 6.20		
Past savings	58	3.38 ± 3.77		
Other	31	4.71 ± 6.48		
Subjective well-being			38.99	*P* < 0.05
Very unhappy	31	11.48 ± 8.06		
Rather unhappy	119	7.44 ± 6.27		
Neutral	396	4.99 ± 5.32		
Rather happy	691	3.51 ± 4.68		
Very happy	309	2.91 ± 4.59		
Suffer from chronic disease			8.74	*P* < 0.05
Yes	1,145	4.80 ± 5.63		
No	401	2.60 ± 3.77		
Visual Condition			106.36	*P* < 0.05
NO Fuzzy	265	1.83 ± 3.30		
A bit	805	3.47 ± 4.27		
Fuzzy	476	6.85 ± 6.58		
Smoking Status			16.74	*P* < 0.05
No smoking	1,131	4.70 ± 5.57		
Former Smoking	112	2.79 ± 3.03		
Smoking	303	3.02 ± 4.57		
Drinking Status			19.94	*P* < 0.05
No Drinking	1,072	4.74 ± 5.54		
Former Drinking	101	4.22 ± 5.24		
Drinking	373	2.76 ± 4.21		
Labor Intensity			12.76	*P* < 0.05
Very light	548	4.22 ± 5.23		
Lighter	495	4.48 ± 5.72		
Generally	359	3.79 ± 4.81		
Heavy	130	4.56 ± 5.27		
Very heavy	14	3.71 ± 3.79		
Physical examination			6.09	*P* < 0.05
Yes	1,242	4.55 ± 5.55		
No	304	2.91 ± 3.82		
Social support level			30.97	*P* < 0.05
Low	53	7.38 ± 7.55		
Medium	1,177	4.57 ± 5.48		
High	316	2.44 ± 3.29		
Depressed			28.34	*P* < 0.05
Yes	648	4.03 ± 5.06		
No	898	4.51 ± 5.60		
Sleep quality			13.31	*P* < 0.05
Sleep disorders	738	6.04 ± 6.17		
No sleep disorders	808	2.57 ± 3.63		
Coarse grains consumption			11.11	*P* < 0.05
Not eating coarse grains	613	5.00 ± 5.69		
1–2 times/week	481	3.49 ± 4.66		
3–5 times/week	240	3.27 ± 4.26		
Every day	212	4.76 ± 6.70		
BMI Levels			2.75	*P* < 0.05
Lean	136	3.93 ± 5.50		
Normal	724	4.00 ± 5.08		
Overweigh	494	4.26 ± 5.16		
Obesity	192	5.20 ± 6.14		
Self-rated Health			−17.326	*P* < 0.05
Healthy	672	1.93 ± 3.21		
Unhealthy	874	6.00 ± 5.87		

### Multiple linear regression of anxiety scores in rural older adults

Taking the anxiety score of the rural older adults as the dependent variable, multiple linear regression analysis was conducted with the following statistically significant independent variables: age, sex, marital status, educational level, primary source of financial support, subjective well-being, chronic disease status, visual acuity, smoking status, drinking status, coarse grain consumption, labor intensity, physical examination, social support level, sleep quality, body mass index (BMI), and self-rated health. The results indicated that age, marital status, visual condition, physical examinations, self-rated health, sleep quality, depression, coarse grain consumption, subjective well-being, and social support level were key factors influencing anxiety levels among the rural older adults population (Table 2).

**Table 2:** Multiple regression analysis of anxiety scores in rural older adults (*N* = 1,546).

Variable	*SE*	Beta	*t*	*P*	*b*(95%CI)
Constant	3.046		3.711	*p < 0.001*	(1.436, 4.655)
Age (60–69)					
70–79	−0.027	0.23	−1.264	*0.206*	(−0.741, 0.160)
80 and above	0.323	−0.103	−4.468	*P < 0.001*	(−2.076, −0.809)
Gender (male)	0.248	0.036	1.565	0.118	(−0.098, 0.874)
Educational level (Illiterate)	0.216	−0.005	−0.236	0.814	(−0.474, 0.372)
Marital status (not married)	0.241	0.101	5.058	*P < 0.001*	(0.747, 1.694)
Subjective well-being	0.112	−0.142	−7.208	*P < 0.001*	(−1.023, −0.585)
Primary source of financial support (self-labor)					
Child support	0.267	−0.002	−0.091	0.927	(−0.549, 0.500)
Government subsidies	0.301	0.066	2.841	0.005	(0.264, 1.444)
Pasting Savings	0.535	0.014	0.705	0.481	(−0.673, 1.428)
Other	0.707	0.014	0.756	0.450	(−0.853, 1.922)
Suffering from chronic diseases (No)	0.232	0.032	1.663	0.096	(−0.069, 0.840)
Visual condition	0.158	0.109	5.377	*P < 0.001*	(0.538, 1.156)
Smoking status (no smoking)					
Former Smoking	0.454	−0.079	−3.564	*P < 0.001*	(−2.506, −0.727)
Smoking	0.292	−0.021	−0.977	0.329	(−0.859, 0.288)
Drinking status (no drinking)					
Former drinking	0.475	−0.001	−0.029	0.977	(−0.945, 0.918)
Drinking	0.273	−0.027	−1.241	0.215	(−0.876, 0.197)
Coarse grains consumption	0.094	−0.038	−2.017	0.044	(−0.375, −0.005)
Labor Intensity (very light)					
Lighter	0.250	−0.024	−1.068	0.286	(−0.757, 0.223)
Generally	0.295	−0.009	−0.399	0.690	(−0.697, 0.461)
Heavy	0.422	0.046	2.058	0.040	(0.041, 1.696)
Very heavy	1.038	0.012	0.620	0.535	(−1.392, 2.680)
Physical examination (NO)	0.262	0.051	2.579	0.010	(0.162, 1.189)
Sleep quality (no sleep disorders)	0.210	0.081	4.1089	*P < 0.001*	(0.447, 1.270)
Depressed (No)	0.225	0.479	22.865	*P < 0.001*	(4.703, 5.586)
Self-rated health (unhealthy)	0.219	−0.124	−6.049	*P < 0.001*	(−1.754, −0.895)
BMI level (Lean)					
Normal	0.351	0.008	0.228	0.820	(−0.608, 0.768)
Overweigh	0.365	0.004	0.115	0.909	(−0.674, 0.758)
Obesity	0.423	0.028	1.076	0.282	(−0.375, 1.285)
Social support level	0.232	−0.058	−2.874	0.004	(−1.124, −0.212)

Dependent variable: anxiety scores. Adjusted *R*^2^ = 0.508.

We took *p* < 0.05 as the criterion for significance. The intensity of the difference is indicated by the *t* value or the *F* value. The comparison of two sets of data is shown as the *t* value, and the comparison of three or more sets of data is shown as the *F* value.

### Results of the rural older adults anxiety random forest model

**Figure 1:**
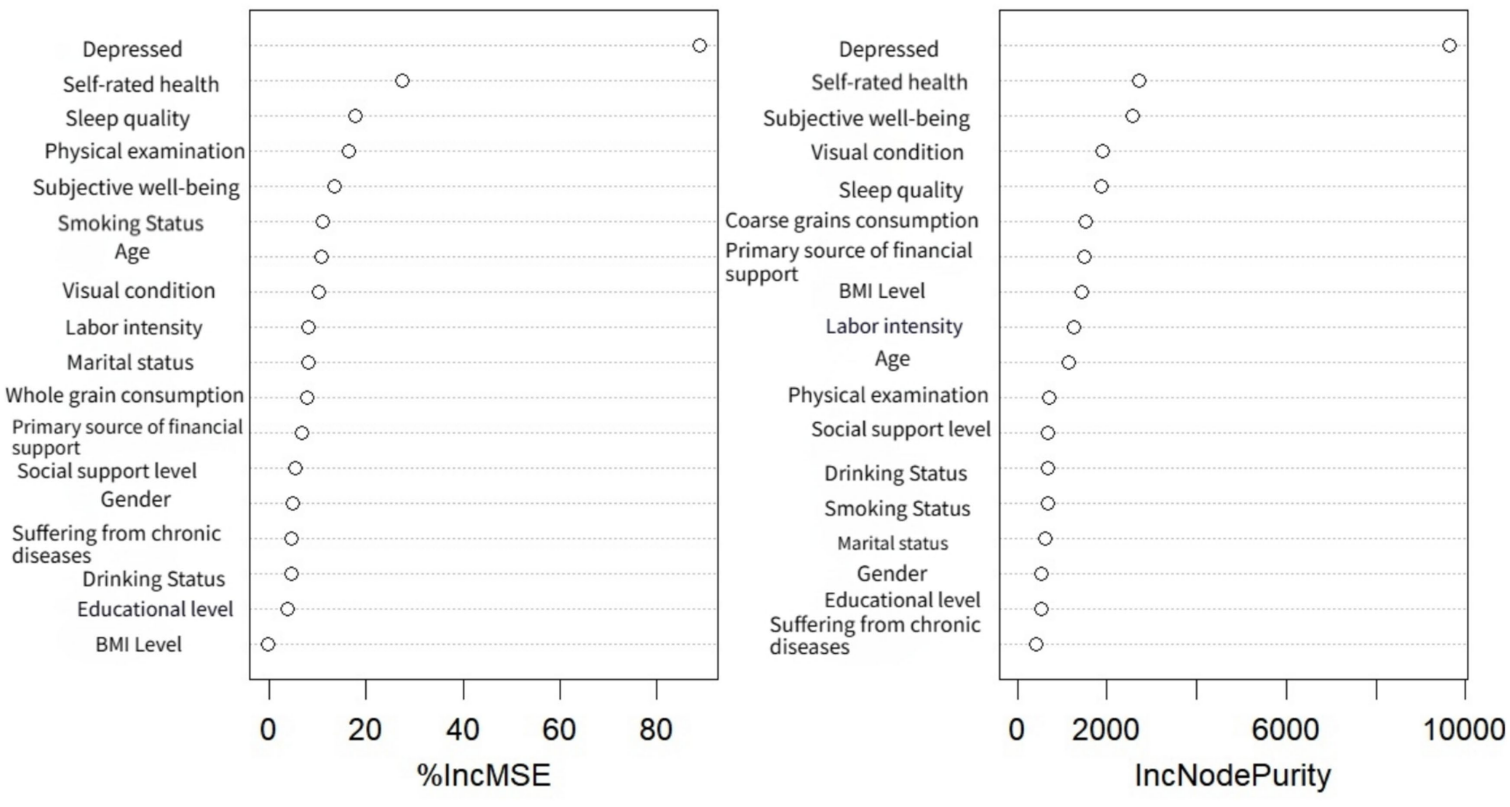
Importance ranking of random forest variables.

RStudio software was utilized to construct a random forest model, with the number of random seeds set to 666. The pre-processed data were randomly partitioned into a training set (75%) and a test set (25%). Subsequently, the random forest model was fitted using the training set. The performance of the model was evaluated using Root Mean Squared Error (RMSE), coefficient of determination (*R*^2^), and Mean Absolute Error (MAE). Generally, higher *R*^2^ values, along with lower RMSE and MAE values, indicate superior model accuracy. The dependent variable in this analysis was the anxiety score of the older adults population, while the remaining variables were incorporated into the random forest model as categorical predictors. Parameter tuning was performed using the caret package for grid search, which identified mtry = 5 as the optimal parameter. Consequently, a random forest model was constructed with ntree = 500 and mtry = 5. Under these conditions, the random forest model achieved an R^2^ of 0.484, RMSE of 3.589, and MAE of 2.581, demonstrating satisfactory performance. The variable assignments for the random forest model are presented in Table 3, while the importance of all explanatory variables is illustrated in Table 4 and Figure 1. As shown in Table 4, the top IncMSE and IncNodePurity values for depression, self-rated health, subjective well-being, sleep quality, and vision closely aligned with the results obtained from multiple linear regression.

**Table 3:** Random forest model assignment.

Variable	Assignment mode
Age	60–69 = 0; 70–79 = 1; 80及以上 = 2
Gender	Male = 1; Female = 2
Primary source of financial support	Self-labor = 1; Child support = 2; Government subsidy = 3; Past savings = 4; Other = 5
Educational level	Illiterate = 0; Literate = 1
Subjective well-being*	Very unhappy = 1; Rather unhappy = 2; Neutral = 3; Rather happy = 4; Very happy = 5
Marital status	Married = 1; Not married = 0
Suffering from chronic diseases	Yes = 1; No = 0
Smoking Status	No-Smoking = 1; Former Smoking = 2; Smoking = 3
Drinking Status	No-Drinking = 1; Former Drinking = 2; Drinking = 3
Coarse grains consumption*	Not eating coarse grains = 1; 1–2 times/week = 2; 3–5 times/week = 3; Almost every day = 4
Labor Intensity*	Very light = 1; Lighter = 2; Generally = 3; Heavier = 4; Very heavy = 5
Physical examination*	Yes = 1; No = 2
Sleep quality	Sleep disorders = 1; No sleep disorders = 0
Depressed	Yes = 1; No = 0
Social support level	Low = 0; Medium = 1; High = 2
Visual condition*	Not fuzzy = 1; a bit = 2; Fuzzy = 3
Self-rated Health	Unhealthy = 0; Healthy = 1
BMI level	Lean = 0; Normal = 1; Overweight = 2; Obesity = 3

*Subjective well-being: overall, would you say you are satisfied with your life?

Visual condition: do things appear fuzzy or out of focus to you?

Coarse grains consumption: do you usually eat coarse grains (such as millet, sweet potato, corn, etc.)?

Labor intensity: how would you describe your usual physical work intensity?

Physical examination: have you undergone any physical examination in the past 12 months?

**Table 4:** Ranking of importance of factors influencing anxiety in rural older adults.

Variable	%IncMSE	IncNodePurity
Depressed	88.911	9653.255
Self-rated Health	27.391	2720.070
Subjective well-being	13.394	2570.139
Visual condition	10.374	1910.284
Sleep quality	17.793	1872.162
Coarse grains consumption	7.774	1537.674
Primary source of financial support	6.907	1489.311
BMI Level	−0.333	1431.961
Labor intensity	8.154	1249.925
Age	10.885	1158.196
Physical examination	16.489	710.944
Social support level	5.428	692.073
Drinking Status	4.607	683.613
Smoking Status	11.098	668.693
Marital status	8.038	620.278
Gender	4.968	548.181
Educational level	3.815	533.425
Suffering from chronic diseases	4.710	411.359

## Discussion

Based on data collected from Anhui Province, China, this study systematically investigated the current prevalence and influencing factors of anxiety among rural older adults individuals aged 60 years and above. The survey revealed that the prevalence rate of anxiety in this population was 36.40%. The findings indicated that age, marital status, subjective well-being, visual condition, physical examination, self-rated health, sleep quality, depression, consumption of coarse grains, and social support significantly influenced anxiety levels. Furthermore, a random forest model was developed to quantitatively assess the relative importance of these factors. The results of the random forest analysis demonstrated that depression, self-rated health, subjective well-being, sleep quality, and visual condition were the most significant contributors to anxiety among the older adults.

Previous research has identified the detrimental effects of depression, self-rated health, and sleep quality on anxiety. Our study further substantiates the negative correlations among depression, self-rated health, sleep quality, and anxiety. Anxiety and depression, as prevalent mental health conditions in the older adults population, are intricately interconnected. Studies have demonstrated that the co-occurrence of depression and anxiety represents the highest incidence rate of mental health outcomes among all combinations of emotional disorders. In this study, the prevalence rates of comorbid depression and anxiety were 41.90 and 30.66%, respectively, which are significantly higher than those reported in prior studies (23). This could potentially be attributed to the varying survey instruments utilized in our study. Prior research has indicated that the GAD-7 and PHQ-9 scales tend to yield higher detection rates (24, 25). Greater emphasis should be placed on addressing the mental health needs of the older adults in the region, with access to professional mental health support being ensured. Self-rated health reflects an individual’s subjective assessment of their own health status. Research has demonstrated that individuals with poor physical health tend to report worse mental health, particularly when compared to peers of the same age group. Those with suboptimal physical health often perceive that their life circumstances and economic conditions place additional burdens on their children and family members, leading to a psychological gap. Additionally, poorer physical health is associated with reduced participation in recreational and social activities, which numerous studies have highlighted as crucial for maintaining mental well-being. The necessity to curtail social activities due to physical limitations may further exacerbate mental health challenges (26, 27). Previous research has demonstrated a negative correlation between anxiety and subjective well-being, with the effects of anxiety and subjective influencing each other reciprocally. During the aging process, older adults undergo significant changes in physiological, psychological, and social domains. Maladaptive individuals often experience substantial psychological stress, leading to strained or indifferent interpersonal relationships. This unhappiness frequently impacts the physiological well-being of older adults, contributing to various physical and physiological illnesses, which in turn severely affect their psychological state and quality of life (28, 29). We observed that poor sleep quality is significantly associated with elevated levels of anxiety, as approximately 47.73% of older adults reported experiencing sleep disorders. Poor sleep quality often co-occurs with anxiety symptoms, which manifest as attenuated forms of sleep-related changes in individuals with anxiety. The relationship between sleep and anxiety is intricate and multifaceted. From a neurophysiological standpoint, sleep deprivation or sleep fragmentation leads to hyperactivation of the amygdala (the brain’s emotional processing center), thereby increasing sensitivity to threatening stimuli and potentially triggering or exacerbating anxiety symptoms (30). Long-term poor sleep quality may indirectly influence the emotional regulation pathway via the release of pro-inflammatory cytokines, potentially leading to the emergence of negative emotions, such as anxiety (31). In addition, poor sleep quality can further exacerbate the decline of other physical functions in the older adults, such as memory impairment, cognitive deterioration, and increase the prevalence of various diseases. This may also lead to reduced mobility and participation in social activities, thereby increasing the risk of anxiety disorders (32). On the other hand, older adults with poor sleep quality tend to exhibit lower levels of mental resilience, which is closely associated with an increased risk of anxiety disorders (33).

Studies have also demonstrated that anxiety is negatively correlated with happiness. During the aging process, older adults individuals undergo significant changes in physiological, psychological, and social aspects. Maladaptive individuals often experience substantial psychological stress, interpersonal tension or indifference, and a lack of happiness, which can adversely affect the physiological well-being of the older adults. This may lead to various physical and mental health issues, thereby severely impacting the psychological state and quality of life of older adults (28). In the existing research on mental health issues among the older adults in China, insufficient attention has been given to the impact of sensory disorders, such as vision impairment, on psychological well-being. Nevertheless, studies have confirmed that sensory disorders, including vision and hearing loss, can lead to an increase in negative emotions among older adults. It is evident that impaired vision is associated with a higher likelihood of depression. A British study involving 13,900 older adults revealed that individuals with visual impairments were more prone to experiencing functional difficulties, which could contribute to depressive symptoms (34). There is limited evidence supporting a significant association between anxiety and visual impairment. A study conducted in Spain demonstrated that individuals with both visual and hearing impairments exhibited a higher prevalence of depression and anxiety compared to those with only hearing impairments, followed by those with solely visual impairments (35). Our study examined the vision status of older adults and identified a statistically significant association between vision status and anxiety in this population. Impaired vision serves as a risk factor for falls among older adults and can further restrict their daily functioning, resulting in various functional challenges, such as difficulty performing daily activities, diminished social interaction, reduced physical activity, and inadequate nutrition. These factors collectively contribute to the development of anxiety (36–39).

Another interesting finding of this study was the negative association between coarse grain consumption and anxiety. In recent years, the relationship between dietary factors and anxiety has garnered increasing attention. We hypothesize that the consumption of whole grains may alleviate anxiety in older adults populations through the following mechanisms. Firstly, inflammation and insulin resistance are well-established biochemical mechanisms underlying emotional impairment and anxiety. The intake of whole grain foods is associated with improved postprandial glucose metabolism, positively influencing insulin sensitivity and effectively regulating glucose-related mood changes (40). Secondly, whole grain foods, such as various types of whole grains, are highly nutritious and rich in betaine, vitamin B6, vitamin B12, and folate. These components are integral to the individual’s 1-carbon metabolism cycle, which has been shown to be associated with the regulation of negative emotions (41). Finally, research indicates that whole grain consumption is correlated with other healthy lifestyle practices, which might contribute to the reduced anxiety levels observed in older adults who consume coarse grains (42).

In addition, the findings of this study regarding the association between marital status, social support, and anxiety among the older adults are consistent with prior research. In this study, social support did not demonstrate a significant influence, which may be attributed to the extremely low proportion (3.4%) of older adults who reported low levels of social support. This limited prevalence might have diminished the impact of social support on anxiety in older adults. Furthermore, we observed that the likelihood of anxiety decreased with advancing age, a finding that contrasts with earlier studies (43, 44). We hypothesize that this phenomenon is associated with the evolving social roles of older adults. From the perspective of their social and familial roles, younger seniors experience a shorter duration of chronic illness and bear a lesser physical burden. As they transition into older age, they continue to proactively assume responsibilities such as household chores and childcare. Additionally, some individuals entering early old age may choose to extend their social engagement and contribute their remaining energy. This could potentially explain why younger seniors are more susceptible to anxiety (45). On the other hand, this phenomenon may be closely associated with the cultural concepts that are characteristic of rural China. Within the tradition of “respect for the older adults,” older adults hold a position of authority within the family structure and are often revered and cared for by those around them. This social dynamic can help mitigate the anxiety and feelings of marginalization that the older adults might otherwise experience.

### Limitations

Finally, the limitations of this study are outlined as follows. First, prior research has demonstrated that a multitude of factors, including biological, cultural, and environmental influences, interact in the development of anxiety disorders. However, due to data constraints, this study was unable to incorporate all relevant factors comprehensively. Second, as a cross-sectional study, while we identified potential risk factors associated with anxiety, we cannot establish causal relationships between these factors and anxiety disorders. Additionally, this study did not account for the age-specific characteristics of the older adults population in relation to anxiety disorders. Given that old age is characterized by distinct stages—early, middle, and late—it is plausible that the patterns and impacts of factors influencing anxiety disorders may vary across these stages. Lastly, the majority of the data in this study were self-reported by survey participants, which may introduce subjective bias.

## Conclusion

In summary, anxiety disorders among the older adults in rural China are associated with age, marital status, subjective well-being, visual condition, physical examination results, consumption of coarse grains, depression, sleep quality, self-rated health, social support level. This finding corroborates that anxiety in the older adults is a multifactorial outcome. Enhancing the health status of the older adults and increasing their sense of happiness and fulfillment are critical objectives of the Healthy China strategy. Currently, it is feasible to enhance the national dissemination of mental health knowledge in rural areas, intensify interventions for common mental disorders such as depression and anxiety, as well as address psychological and behavioral issues, thereby improving the mental health of rural older adults populations. Simultaneously, efforts should be made to comprehensively disseminate dietary nutrition knowledge, formulate dietary guidelines tailored to the characteristics of different groups, guide rural residents in forming scientifically sound eating habits, and promote the establishment of a healthy dietary culture. In promoting healthy lifestyles and strengthening health education, these initiatives will contribute significantly to the “Healthy China” construction.

## Data Availability

The original contributions presented in the study are included in the article/supplementary material, further inquiries can be directed to the corresponding author.
